# Understanding the management of electronic test result notifications in the outpatient setting

**DOI:** 10.1186/1472-6947-11-22

**Published:** 2011-04-12

**Authors:** Sylvia J Hysong, Mona K Sawhney, Lindsey Wilson, Dean F Sittig, Adol Esquivel, Simran Singh, Hardeep Singh

**Affiliations:** 1 Houston VA Health Sciences Research & Development Center of Excellence, The Center of Inquiry to Improve Outpatient Safety Through Effective Electronic Communication, Michael E. DeBakey Veterans Affairs Medical Center and the Section of Health Services Research, Department of Medicine, Baylor College of Medicine, Houston, Texas, USA; 2 University of Texas School of Biomedical Informatics and the UT-Memorial Hermann Center for Healthcare Quality & Safety, Houston, Texas, USA; 3 St. Luke's Episcopal Health System, Houston, Texas, USA; 4 Louis Stokes Cleveland VA Medical Center, Cleveland, Ohio, USA

**Keywords:** Decision Support Systems, Clinical, Automated notification, diagnostic errors, abnormal diagnostic test results, Medical Records Systems, Computerized, patient follow-up, patient safety, health information technology, communication, primary care

## Abstract

**Background:**

Notifying clinicians about abnormal test results through electronic health record (EHR) -based "alert" notifications may not always lead to timely follow-up of patients. We sought to understand barriers, facilitators, and potential interventions for safe and effective management of abnormal test result delivery via electronic alerts.

**Methods:**

We conducted a qualitative study consisting of six 6-8 member focus groups (N = 44) at two large, geographically dispersed Veterans Affairs facilities. Participants included full-time primary care providers, and personnel representing diagnostic services (radiology, laboratory) and information technology. We asked participants to discuss barriers, facilitators, and suggestions for improving timely management and follow-up of abnormal test result notifications and encouraged them to consider technological issues, as well as broader, human-factor-related aspects of EHR use such as organizational, personnel, and workflow.

**Results:**

Providers reported receiving a large number of alerts containing information unrelated to abnormal test results, many of which were believed to be unnecessary. Some providers also reported lacking proficiency in use of certain EHR features that would enable them to manage alerts more efficiently. Suggestions for improvement included improving display and tracking processes for critical alerts in the EHR, redesigning clinical workflow, and streamlining policies and procedures related to test result notification.

**Conclusion:**

Providers perceive several challenges for fail-safe electronic communication and tracking of abnormal test results. A multi-dimensional approach that addresses technology as well as the many non-technological factors we elicited is essential to design interventions to reduce missed test results in EHRs.

## Background

The American Recovery and Reinvestment Act of 2009 (ARRA) awards up to $63,750 of incentive payments to health care providers who demonstrate "meaningful use" of a "qualified electronic health record" (EHR), and will eventually penalize providers who do not demonstrate such meaningful use by 2015[1]. One important aspect of the meaningful use concept is the application of clinical decision support (CDS) tools to improve coordination and quality of health care[2]. For instance, real-time electronic notification of abnormal test results via the EHR may facilitate timely follow-up, particularly in outpatient settings, where many results are not immediately life threatening and not verbally reported to ordering clinicians[3,4]. Outpatient test results are especially vulnerable to "falling through the cracks" [5,6] and are often cited as reasons for delays in diagnosis and treatment, patient harm and malpractice claims[7-14].

To achieve meaningful use of EHRs as envisioned by the federal government, providers need to be proficient in use of the decision support features available in their EHR and understand how they fit into the clinical workflow. However, provider needs and the current workflow are not always considered when designing EHR systems[15]. In many EHR systems, an electronic notification feature (e.g., the "View Alert" window used by the U.S. Department of Veterans Affairs' (VA) Computerized Patient Records System (CPRS), or the "In-Basket" feature available in Epic's EpicCare EHR) delivers test results to a message inbox that providers can access after they login to the EHR. (see Additional file 1). Alerting through this asynchronous mechanism is quite different from "synchronous" CDS alerts such as drug-drug interaction (DDI) alerts, which interrupt users when they are entering medication orders. While synchronous alerting has been studied quite extensively, [16-20] alerting through "asynchronous" channels has received little attention. Unlike actions related to DDI alerts, follow-up actions required of test results alerts are not necessarily required immediately after alert delivery. How these asynchronous alerts integrate into a provider's workflow is largely unknown.

We recently examined providers' responses and follow-up actions on over 2500 alerts of abnormal test results in CPRS[5,6]. Of these, we found providers did not acknowledge (i.e., did not read) 18.1% of alerts pertaining to abnormal imaging results and 10.2% of abnormal laboratory alerts. Furthermore, approximately 8% of abnormal imaging and 7% of abnormal laboratory results lacked timely follow-up at 30 days. We also found that there was no significant relationship between acknowledging an alert and timely follow-up. Thus, despite delivery of test results directly to a clinician's View Alert window, abnormal results did not always receive timely follow-up.

Clinicians do not optimally utilize all of the functions in the EHR; for instance, we found that about half (46%) of clinicians did not use the specific features of the View Alert window that facilitate better processing of electronic alerts[21]. Instead, providers often used handwritten notes or external systems (e.g., ticklers on their calendar) to help process their alerts[21]. Thus, many factors beyond the technology itself will likely predict how "meaningfully" providers will use CDS tools for test result reporting in the future[22,23].

To obtain a comprehensive understanding of the management of test result alerts in EHRs and to explain why abnormal results might be missed, we used a qualitative, sociotechnical, systems-based approach. Our objective was to conduct a qualitative study at two large VA facilities to understand barriers, facilitators, and potential interventions for effective and safe management of abnormal test results delivered through the EHR. We relied on human factors engineering principles to frame our research questions and explore issues beyond the confines of the computer.

## Methods

### Human Subjects

This study was approved by the Baylor College of Medicine Institutional Review Board for compliance with accepted human subject research practices consistent with the Helsinki Declaration (Protocol # H-21817, *Improving Outpatient Safety Through Effective Electronic Communication*).

### Design and Setting

We conducted three focus groups at each of two large, geographically dispersed VA medical centers between January and May 2009. Focus groups are ideally suited for this type of research because they allow for live interaction among participants, and richer data than what survey methods could elicit[24]. Table 1 presents basic characteristics for the two sites.

**Table 1:** Basic characteristics of participating sites and focus group composition.

Site Characteristics		Site A	Site B
Number of Patients enrolled		122,452	82,000
Outpatient visits/year		815,695	780,000
Academically affiliated?		Yes	Yes
Number of Primary Care Providers		38	16

**Focus Group Composition**			

FG No.	Role	**Site A**	**Site B**

1	Lab/Radiology Personnel	2	0
	IT Personnel	1	1
	Primary Care Providers	3	8
	Specialist	1	0

2	Lab/Radiology Personnel	1	0
	IT Personnel	1	0
	Primary Care Providers	4	8
	Specialist	0	0

3	Lab/Radiology Personnel	1	0
	IT Personnel	1	0
	Primary Care Providers	4	8
	Specialist	0	0

Total		19	25

For almost a decade, both study sites have used the Computerized Patient Record System (CPRS), the EHR system in use at VA facilities nationwide. Within CPRS, providers are notified of test results in a "View Alert" window that is displayed when a provider logs in. Although some functionality is configurable at the facility and user levels, most software changes to CPRS are made at the national level and disseminated simultaneously to all VA facilities. Consequently, CPRS configurations are far more standardized among facilities than most other commercially available EHR systems.

### Participants and Sampling Frame

Forty-four full-time personnel representing primary care, radiology, information technology (IT) and laboratory services participated in the focus groups. We purposively selected participants from these fields specifically because their job responsibilities involved considerable interaction with test result notifications in the View Alert window at different parts of the workflow. Additionally, the primary care providers (PCPs, which consisted of physicians and physician assistants) were purposively sampled based on previous analyses [5] for having a high or a low number of alerts lost to follow-up within a 30 day period. Groups were limited to the recommended size of six to eight participants[24]. Table 1 presents the composition of each focus group by staff specialty.

### Procedure

Details of our data collection and analysis procedures are published elsewhere [25] and are summarized here. Participants were recruited via phone/email, and signed an informed consent form before participating in the focus groups after having the opportunity to ask questions about the study, including issues of anonymity and confidentiality.

During the first two focus groups, we asked participants to discuss barriers and facilitators to successful management and follow-up of abnormal test result alerts and provide suggestions for improvement. We encouraged participants to think beyond the CPRS user interface, software, and hardware, and to consider broader human factors engineering issues such as organizational, personnel, workflow, and work environment concerns. Participants in the third focus group at each site concurred or dissented with the most frequently raised themes from the first two focus groups and discussed further barriers and suggestions for improvement.

### Data Analysis

**Figure 1:**
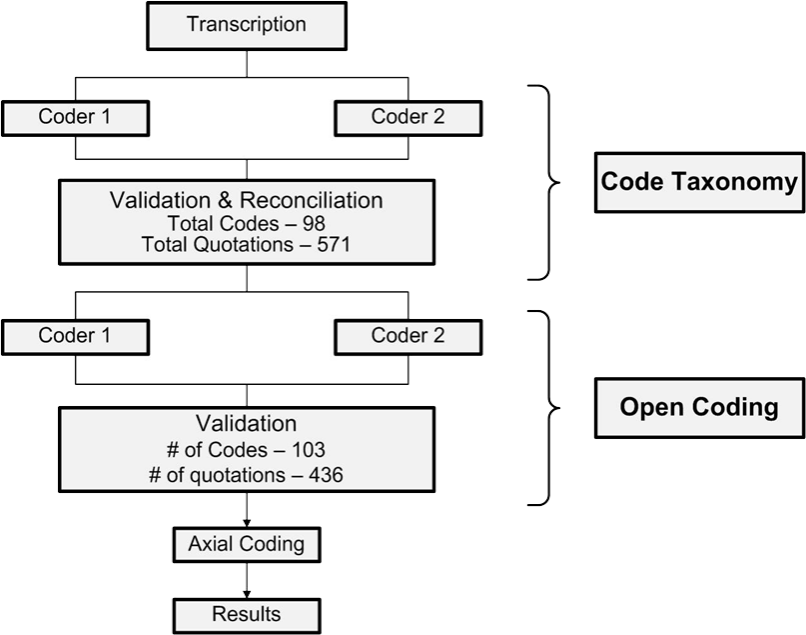
**Summary of coding process and analysis flow**.

We used thematic analysis [26] to analyze our focus group transcripts, with the goal of identifying common alert management barriers and facilitators and suggestions for improving the alert system. Analysis tasks included a) the development of an initial coding taxonomy; b) open coding, in which text passages were examined for recurring themes and ideas; and c) axial coding, in which themes were organized into meaningful relationships. Figure 1 depicts the flow of analysis tasks.

#### Taxonomy Development and Open Coding

Two coders with qualitative research experience independently coded the focus group transcripts for content pertaining to barriers, facilitators, and suggestions for improvement. The coded data sets were then merged and reviewed by a third coder (the validator) to reconcile nearly identical quotations, codes carrying different labels yet referring to the same phenomenon, and codes needing further discussion to reach consensus. The coding team then met to review and reach agreement on discrepant codes and quotations. After a one-week waiting period to reduce priming effects, each coder independently coded the finalized quotation list using the newly developed taxonomy. The validator again identified inter-coder discrepancies, which were resolved by consensus.

#### Axial Coding

We first organized coded passages according to groundedness (i.e., the number of quotations to which a code was assigned) to determine the most commonly cited barriers, facilitators, and suggestions for improvement. We then compared the patterns of coded passages by site and used these comparisons to identify larger, recurring themes.

## Results

**Figure 2:**
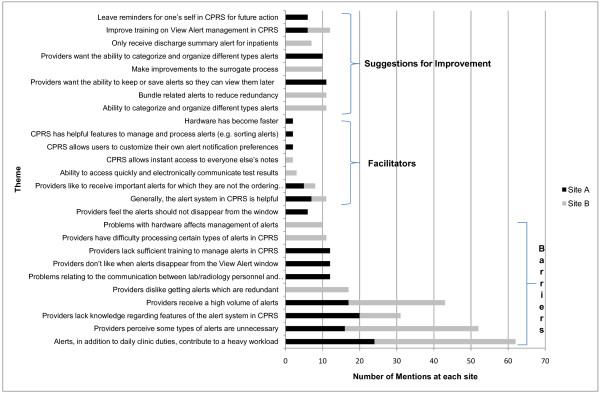
**Most commonly cited barriers, facilitators, and suggestions for improvement, by site**.

Figure 2 presents the barriers, facilitators, and suggestions for improvement that were most frequently raised by participants according to their groundedness; all themes presented were mentioned by multiple participants. The most commonly cited barriers overlapped considerably across sites and focus groups, despite differences in site characteristics and focus group composition. Furthermore, these themes were raised by multiple participants across the focus groups, suggesting they were not simply an artifact of a single, dominant participant.

### Number of Alerts Received

The most frequently raised barrier was the number of alerts received by providers. In addition to test result alerts providers received many other types of notifications, which complicated the task of reviewing test results and providing timely follow-up care. Participants expressed concern about both the total number of alerts and the proportion of notifications perceived as unnecessary. Providers in all focus groups reported that their already heavy clinical workloads left very little time for the task of alert management:

"On an average it takes about two to three minutes per alert. And we get sixty to seventy alerts per day. So, there's no time allowed for alerts... I've just finished seeing patients. I have to go back and handle all the alerts. Some people actually come in on weekends. So, yeah, time is definitely a factor." -PCP, Site A

"One of the issues is just the sheer volume of alerts, and there are a number of alerts that in all honesty [you] really don't have any business seeing." -PCP, site B

"I counted 150 alerts one day just to see how many were coming in that normal day, and this is a fairly regular day, 150 alerts. That's a lot of time spent trying to go through that while you're seeing patients, while there's no in between time to get caught up." -PCP, Site B

Types of alerts perceived as unnecessary varied across the sites, but at both sites providers discussed situations when they were needlessly notified of events they deemed as strictly "for your information." These included, for example, overly detailed status updates of services performed outside primary care:

"The surgeon needs to take care of his own alerts. I don't need to be a backup for him. I mean, you know, he's licensed, right? He holds a license. He needs to worry about his license. He needs to take care of his stuff. And if every department did that, I mean, that would cut down our workload by fifty percent. That's where the problem is, everybody expects us to be the backup, and there's really no need." PCP, Site A

"You could have half a dozen notifications on a given consult which really are unimportant. The only thing I really need to know about is if it was actually scheduled and what the date was in case it's something I want to have done soon, and this is way too far away, or if it's canceled altogether. I really don't care about any of the other notifications, all these notes that they pass back and forth about whether or not they contacted the patient, whether or not he had transportation." PCP, Site B

**Figure 3:**
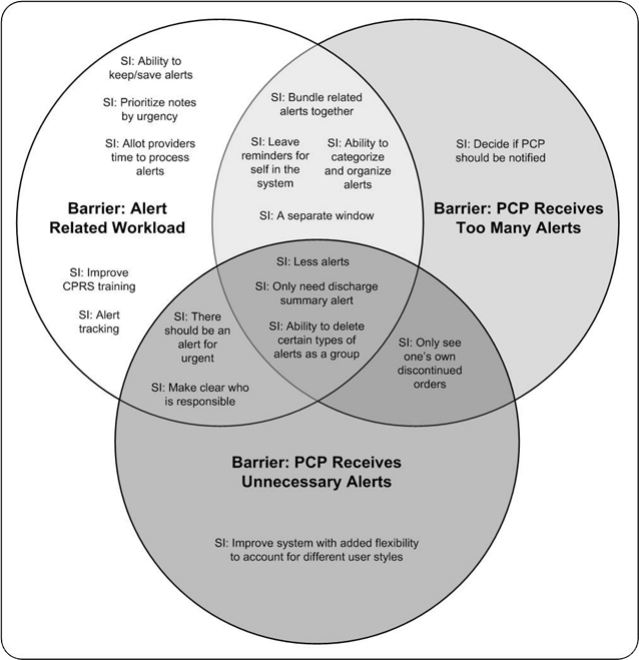
**Co-occurrence analysis of participants' proposed solutions to volume-related barriers**.

To further explore the problem of large numbers of alerts, we conducted a co-occurrence analysis to examine common themes in participants' proposed solutions. We identified all passages in which suggestions for improvement co-occurred with any of the three quantity-related barriers most heavily discussed in the focus groups: too many alerts, unnecessary alerts, and an overly heavy patient-related workload created by the alerts. As seen in Figure 3, the three barriers co-occurred with a total of 17 suggestions for improvement. Fourteen were associated with overcoming the workload barrier; these suggestions involved both changes to CPRS (e.g., ability to categorize alerts, bundling alerts together) and changes to workflow (e.g., allocating protected time to manage alerts). Ten of seventeen suggestions applied to multiple barriers, suggesting that these barriers are interrelated. Interestingly, only three suggestions were uniquely associated with the two barriers about number of alerts. This analysis suggests that workload created by alerts is a complex barrier needing multidimensional solutions.

### Tracking and Categorizing Relevant Clinical Information

Another salient theme was providers' desire for a mechanism within CPRS to organize, track, and retrieve alerts so that providers remember to follow up on needed care. As the CPRS View Alerts system was designed to *alert *providers so they could take action at the time of the alert, no functions for longitudinal tracking currently exist in CPRS. Therefore, at both sites, better EHR capabilities to help visualize, organize, and track alerts ranked among the most frequently cited suggestions.

I always wished to see a way to see or a way to know because then I can know how they're performing. There is no way you can tell me how many consults you've placed to GI in the month of November. There's no place I can click... . (PCP, Site A)

### Provider Knowledge of EHR Features

Notably, providers often requested to add functionality to CPRS that already existed (e.g., sorting, ability to delete multiple alerts as a batch), suggesting they lacked knowledge about features within CPRS to manage the large number of incoming alerts:

"Oh, another thing, I learned yesterday that you can do it [sort] by patient also, so mine were all mixed up. So, I just learned that today before coming to this meeting that you can [sort], did you know that? ... That's something which I learned today after eight years of being at the VA."

-PCP, Site A

Along similar lines, participants strongly advocated improvements in CPRS training to highlight existing EHR features specific to alerts citing the existing training sessions they received as "*pretty lackluster*" (PCP, site B).

### Improvements to Alert Content

Finally, providers at both sites also suggested improvements to alert content, such as minimizing inpatient alerts to receiving only the discharge summary, bundling related alerts to avoid overlap and redundancy):

I just want to be alerted, if there's any abnormal I don't care if it's CBC or Chem 7 just say "abnormal lab" and this is the name of the patient, you know, abnormal lab. IF the CBC came in later, click me again- abnormal lab. But if they come in all at the same time don't give me three alerts for the same patient! It clutters my view alert. It's so difficult for me to get into what is important and what is not. (PCP, Site B)

## Discussion

We employed a human factors approach to identify barriers, facilitators, and potential interventions for safe and effective management of EHR-based abnormal test result alerts at two large VA facilities. Providers reported the biggest barrier to be the large number of several other types of alerts that they receive in conjunction with test result alerts; these additional non-test result related alerts were not always high priority and did not always contain urgent information; many were believed to be unnecessary. The total number of alerts was also perceived as too high. These barriers were compounded by the lack of proficiency in alert management features in the EHR. Providers' suggestions to improve follow-up of test results included redesigning the computer interface to improve alert display and allow improved tracking of critical alerts, redesigning clinical workflow, and streamlining policies and procedures related to test result reporting.

A human factors approach has several advantages to understand and improve test result reporting in the EHR[22]. Because of significant previous patient safety concerns regarding follow-up of test results in both paper and EHR based systems,[10,11,27-29] this approach has a higher likelihood of achieving better outcomes as EHRs are implemented widely. Other studies have found similar barriers to the use of computerized clinical reminders (such as for preventive health care), which resemble "non-interruptive" alert notifications in some respects. For example, Patterson and colleagues[30] reported that factors such as workload, the inapplicability of reminders to certain situations, and limited knowledge and training, limited providers' use of reminders. Saleem and colleagues[31] similarly observed that workload and poor usability in the user interface were barriers to effective clinical reminder use. Finally, Campbell and colleagues identified additional new work for clinicians as an unintended consequence of CPOE implementation[32]. Our study adds to this body of work by demonstrating the need for a multi-dimensional approach to address test reporting challenges within the EHR.

### Implications

#### EHR-based test reporting needs improvement to prevent missed results

Currently, EHR-based test result notification systems do not offer an effective way to safely and effectively present critical information such as that related to abnormal test results. Reducing the total number of alerts and removing unnecessary alert types will alleviate this problem. Our findings suggest the need for better interface design to visualize certain types of alerts more effectively, such as those with a higher priority (e.g. critical alerts). Previous work shows that warnings (alerts) are useful only when they communicate information of high importance [33] and overload of information can lead to alert fatigue[34]. Our findings also suggest the need for careful consideration by key decision makers of what should constitute an "alertable" event. Populating the list of alert categories in EHRs judiciously, while not as simple as it seems, is perhaps one of the first steps to address this issue.

#### EHR systems need better tracking capabilities to prevent missed results

Providers voiced several concerns about the current inability to save, track, and retrieve alerts. Information contained in the alerts cannot be retrieved easily once alerts are processed. Currently, longitudinal tracking capabilities such as those that ensure fail-safe follow-up of alert notifications are also lacking. Tracking was raised as a particular concern because there are no current means in this EHR to assist providers in monitoring a patient's progress. For instance, there is no mechanism to remind providers to check on whether a patient has completed a follow-up evaluation in a timely manner. In the absence of these tools, providers used workarounds such as paper-based reminders or leaving progress notes unsigned so as to generate alerts to themselves in the View Alert window. Our findings thus suggest the need for tracking functionality *integrated with *the alert system, akin to that currently available in most email tools (e.g., delivery/read receipts, hierarchical folder structure to store, categorize and retrieve alerts for future reference, or to do lists with past due reminders). Better tracking and retrieval features in future EHRs may significantly improve test result management.

#### Reducing missed test results in EHRs requires a multidisciplinary approach

**Figure 4:**
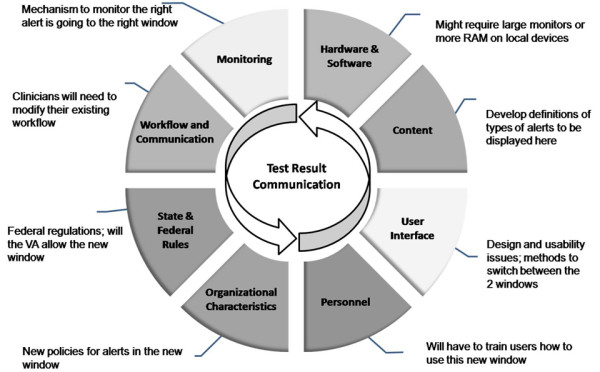
**Example of implementing an intervention using a multidimensional approach: an additional window to better visualize "critical" notifications**.

We recently proposed eight dimensions of safe and effective use of EHRs, many of which go beyond the technology itself: content, software and hardware, user interface, personnel, communication and workflow, organizational policies and procedures, state and federal regulations, and monitoring[35]. Examples in the context of electronic alerts include improving provider training *(personnel)*, changing and improving awareness of policies regarding responsibility for follow-up (*organizational policy*), reducing redundant alerts (c*ontent*), adding functionality for saving, tracking, and retrieving previous alerts (*software*), and improving existing functionality to categorize, organize, and process alerts (*interface*). Most interventions will involve more than one of these dimensions. As illustrated in Figure 4, a seemingly straightforward interface change, such as adding a separate window for visualizing critical alerts, requires consideration of all eight dimensions.

Current legislative decisions have largely focused on the technological aspects of the EHR to improve the quality of health care. However, adopting and implementing meaningful use of an EHR requires more than designing software with good information exchange capabilities as required by the ARRA. Providers will not benefit from technological advances unless a multidisciplinary approach is used to design and implement these systems. For providers to make more meaningful, safe, and effective use of EHR features such as test reporting features, all stakeholders (designers, vendors, organization, and users) must address many socio-technical issues.

### Limitations

Our study has several limitations. Our results may currently only be applicable to the VA's EHR. However, this EHR is used widely and assists over 150,000 clinicians to deliver health care to over eight million veterans[36]; many of its features are present in other commercially available EHRs. Additionally, our multidisciplinary approach is applicable to several test result reporting processes that are likely to be shared among EHRs. Therefore, our findings should be broadly applicable to many organizations currently deciding how to best design and implement technology-based tools to facilitate timely follow-up of abnormal test results.

Second, though we spoke with 44 different participants, the focus groups occurred at only two sites. Future research may need to include a larger number and variety of facilities to rule out the possibility of facility idiosyncrasies as potential confounders.

## Conclusion

We found that providers perceive several challenges for fail-safe electronic communication and tracking of abnormal test results in a state-of-the-art EHR. A multi-dimensional socio-technical approach that includes addressing organizational, personnel, and workflow-related factors in addition to improving technology, is essential to design interventions that help reduce missed test results in EHRs and increase their meaningful use.

## Competing interests

The authors declare that they have no competing interests.

## Authors' contributions

SH - is the study's qualitative core lead; she designed the methodological and analytic strategy for the focus groups; facilitated the focus groups at one site; led the data analysis, and had principal writing responsibility for this manuscript.

MS - conducted data analysis, and materially edited this manuscript.

LW - helped coordinate the focus groups at one site and conducted the data analysis

DS - provided expertise on clinical informatics, helped interpret findings, and materially edited this manuscript.

AE -- provided expertise on clinical informatics, helped analyze findings, and wrote material portions of this manuscript

SS - coordinated and facilitated the focus groups at the alternate site, and had editing responsibility for this manuscript.

HS - is study's principal investigator; he was responsible for the overall design and supervision of this study and the medical record reviews that resulted in sampling classifications. He co-facilitated the focus groups at one of the sites, and materially edited this manuscript.

All authors read and approved the final manuscript

## Pre-publication history

The pre-publication history for this paper can be accessed here:

http://www.biomedcentral.com/1472-6947/11/22/prepub

## Supplementary Material

Additional File 1 **Appendix - CPRS View Alert Window**. Screen shot of the CPRS View Alert Window as seen by the provider. Click here for file

## References

[B1] American Recovery and Reinvestment Act of 2009 2009 Pub.L. 111-5. 1-4-2010.

[B2] Department of Health and Human Services Health Information Technology: Initial Set of Standards, Implementation Specifications, and Certification Criteria for Electronic Health Record Technology; Interim Final Rule 75 2013 2047 1-13-2010. 20344863

[B3] Poon EG Wang SJ Gandhi TK Bates DW Kuperman GJ Design and implementation of a comprehensive outpatient Results Manager J Biomed Inform 2003 36 80 91 10.1016/S1532-0464(03)00061-3 14552849

[B4] Singh H Arora H Vij M Rao R Khan M Petersen L Communication outcomes of critical imaging results in a computerized notification system J Am Med Inform Assoc 2007 14 459 466 10.1197/jamia.M2280 17460135 PMC2244901

[B5] Singh H Thomas E Mani S Sittig D Arora H Espadas D Timely Follow-Up of Abnormal Diagnostic Imaging Test Results in an Outpatient Setting: Are Electronic Medical Records Achieving Their Potential? Arch Intern Med 2009 169 1578 1586 10.1001/archinternmed.2009.263 19786677 PMC2919821

[B6] Singh H Thomas E Sittig DF Wilson L Espadas D Khan M Automated Notification of Abnormal Laboratory Test Results in an Electronic Medical Record: Do Any Safety Concerns Remain? Am J Med 2010 123 238 244 10.1016/j.amjmed.2009.07.027 20193832 PMC2878665

[B7] Gandhi TK Fumbled handoffs: one dropped ball after another Ann Intern Med 2005 142 352 358 15738454 10.7326/0003-4819-142-5-200503010-00010

[B8] Graber ML Franklin N Gordon R Diagnostic error in internal medicine Arch Intern Med 2005 165 1493 1499 10.1001/archinte.165.13.1493 16009864

[B9] Gandhi TK Kachalia A Thomas EJ Puopolo AL Yoon C Brennan TA Missed and delayed diagnoses in the ambulatory setting: a study of closed malpractice claims Ann Intern Med 2006 145 488 496 17015866 10.7326/0003-4819-145-7-200610030-00006

[B10] Hickner J Graham DG Elder NC Brandt E Emsermann CB Dovey S Testing process errors and their harms and consequences reported from family medicine practices: a study of the American Academy of Family Physicians National Research Network Qual Saf Health Care 2008 17 194 200 10.1136/qshc.2006.021915 18519626

[B11] Schiff GD Introduction: Communicating critical test results Jt Comm J Qual Patient Saf 2005 31 63 5 61 15791764 10.1016/s1553-7250(05)31009-9

[B12] Singh H Sethi S Raber M Petersen LA Errors in cancer diagnosis: current understanding and future directions J Clin Oncol 2007 25 5009 5018 10.1200/JCO.2007.13.2142 17971601

[B13] Singh H Daci K Petersen LA Collins C Petersen NJ Shethia A Missed opportunities to initiate endoscopic evaluation for colorectal cancer diagnosis Am J Gastroenterol 2009 104 2543 2554 10.1038/ajg.2009.324 19550418 PMC2758321

[B14] Singh H Kadiyala H Bhagwath G Shethia A El-Serag H Walder A Using a multifaceted approach to improve the follow-up of positive fecal occult blood test results Am J Gastroenterol 2009 104 942 952 10.1038/ajg.2009.55 19293786 PMC2921791

[B15] Johnson CM Johnson TR Zhang J A user-centered framework for redesigning health care interfaces Journal of Biomedical Informatics 2004 38 75 87 10.1016/j.jbi.2004.11.005 15694887

[B16] Paterno MD Maviglia SM Gorman PN Seger DL Yoshida E Seger AC Tiering drug-drug interaction alerts by severity increases compliance rates J Am Med Inform Assoc 2009 16 40 46 10.1197/jamia.M2808 18952941 PMC2605599

[B17] Judge J Field TS DeFlorio M Laprino J Auger J Rochon P Prescribers' responses to alerts during medication ordering in the long term care setting J Am Med Inform Assoc 2006 13 385 390 10.1197/jamia.M1945 16622171 PMC1513672

[B18] Ko Y Abarca J Malone DC Dare DC Geraets D Houranieh A Practitioners' views on computerized drug-drug interaction alerts in the VA system J Am Med Inform Assoc 2007 14 56 64 10.1197/jamia.M2224 17068346 PMC2215077

[B19] van der Sijs H Aarts J Van Gelder T Berg M Vulto A Turning off frequently overridden drug alerts: limited opportunities for doing it safely J Am Med Inform Assoc 2008 15 439 448 10.1197/jamia.M2311 18436915 PMC2442265

[B20] van der Sijs H Mulder A Van Gelder T Aarts J Berg M Vulto A Drug safety alert generation and overriding in a large Dutch university medical centre Pharmacoepidemiol Drug Saf 2009 18 941 947 10.1002/pds.1800 19579216

[B21] Hysong SJ Sawhney M Wilson L Sittig DF Espadas D Davis T Provider management strategies of abnormal test result alerts: a cognitive task analysis J Am Med Inform Assoc 2010 17 71 77 10.1197/jamia.M3200 20064805 PMC2995633

[B22] Rose AF Schnipper JL Park ER Poon EG Li Q Middleton B Using qualitative studies to improve the usability of an EMR J Biomed Inform 2005 38 51 60 10.1016/j.jbi.2004.11.006 15694885

[B23] Aarts J Ash J Berg M Extending the understanding of computerized physician order entry: implications for professional collaboration, workflow and quality of care Int J Med Inform 2007 76 4 13 10.1016/j.ijmedinf.2006.05.009 16798068

[B24] Barbour R Doing Focus Groups 2008 London: Sage

[B25] Hysong SJ Sawhney M Wilson L Sittig DF Esquivel A Watford M Improving outpatient safety through effective electronic communication: A study protocol Implementation Science 2009 4 10.1186/1748-5908-4-62 19781075 PMC2761849

[B26] Stewart DW Shamdasani PN Rook D Focus Groups: Theory and Practice 2006 2 Sage Publications

[B27] Casalino LP Dunham D Chin MH Bielang R Kistner EO Karrison TG Frequency of failure to inform patients of clinically significant outpatient test results Arch Intern Med 2009 169 1123 1129 10.1001/archinternmed.2009.130 19546413

[B28] Poon EG Haas JS Louise PA Gandhi TK Burdick E Bates DW Communication factors in the follow-up of abnormal mammograms J Gen Intern Med 2004 19 316 323 10.1111/j.1525-1497.2004.30357.x 15061740 PMC1492194

[B29] Poon EG Gandhi TK Sequist TD Murff HJ Karson AS Bates DW "I wish I had seen this test result earlier!": Dissatisfaction with test result management systems in primary care Arch Intern Med 2004 164 2223 2228 10.1001/archinte.164.20.2223 15534158

[B30] Patterson ES Doebbeling BN Fung CH Militello L Anders S Asch SM Identifying barriers to the effective use of clinical reminders: bootsrapping multiple methods Journal of Biomedical Informatics 2005 38 189 199 10.1016/j.jbi.2004.11.015 15896692

[B31] Saleem JJ Patterson ES Militello L Render ML Orshansky G Asch SM Exploring Barriers and Facilitators to the Use of Computerized Clinical Reminders J Am Med Inform Assoc 2005 12 438 447 10.1197/jamia.M1777 15802482 PMC1174889

[B32] Campbell EM Sittig DF Ash JS Guappone KP Dykstra RH Types of unintended consequences related to computerized provider order entry J Am Med Inform Assoc 2006 13 547 556 10.1197/jamia.M2042 16799128 PMC1561794

[B33] Maltz M Meyer J Use of warnings in an attentionally demanding detection task Human Factors 2001 43 217 10.1518/001872001775900931 11592663

[B34] Ash JS Sittig DF Campbell EM Guappone KP Dykstra RH Some unintended consequences of clinical decision support systems AMIA Annu Symp Proc 2007 26 30 18693791 PMC2813668

[B35] Sittig DF Singh H Eight rights of safe electronic health record use JAMA 2009 302 1111 1113 10.1001/jama.2009.1311 19738098

[B36] Office of Policy and Planning Analysis of Unique Veterans Utilization of VA Benefits & Services http://www.va.gov/VETDATA/docs/SpecialReports/uniqueveteransMay.pdf 4-29-2009.

