# Supplementary figures and images for: Understanding the management of electronic test result notifications in the outpatient setting
Source: BMC Med Inform Decis Mak. 2011 Apr 12;11:22. doi: 10.1186/1472-6947-11-22 (PMC3100236; doi:10.1186/1472-6947-11-22)

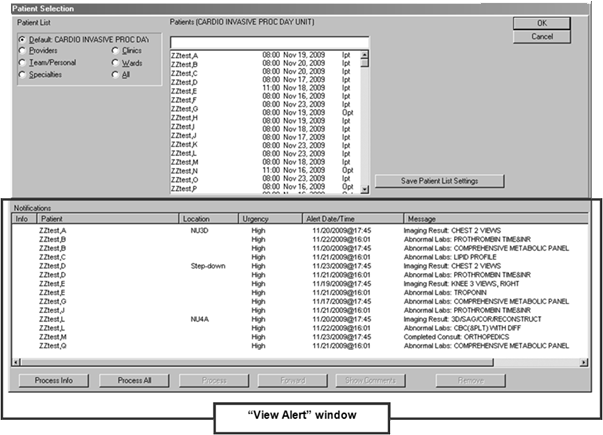

Supplement: Additional File 1 — Appendix - CPRS View Alert Window. Screen shot of the CPRS View Alert Window as seen by the provider. [file 1472-6947-11-22-S1.PNG]
